# Non-resolving, Progressive, Non-ketotic Hyperglycemic Hemichorea-Hemiballismus Syndrome in an Elderly Male

**DOI:** 10.7759/cureus.94603

**Published:** 2025-10-14

**Authors:** Akash Chandani, Cyrine Cunanan, Sumathi Krishnan, Omar Rage

**Affiliations:** 1 Internal Medicine, North Middlesex University Hospital, London, GBR; 2 Geriatrics, North Middlesex University Hospital, London, GBR

**Keywords:** basal ganglia, hemiballismus, hemichorea, movement disorder, non-ketotic hyperglycemia

## Abstract

Non-ketotic hyperglycemic hemichorea-hemiballismus syndrome is a rare neurological condition associated with hyperglycemia from uncontrolled diabetes mellitus. We present a case of a male patient in his late 80s with a background of T2DM who presented with a new onset of progressive involuntary movements involving the left side of his body. Imaging revealed hyperdensity in the right basal ganglia, suggestive of non-ketotic hyperglycemic hemichorea. Management included glycemic control and neuroleptic treatment, with resolution of symptoms in most cases. This case highlights the importance of considering metabolic causes of hemichorea-hemiballismus in elderly patients, glycemic control, and the correlation of these factors with quality of life.

## Introduction

Hemichorea-hemiballismus (HCHB) is a rare neurological disorder characterized by sudden-onset, involuntary, and often violent movements affecting one side of the body. It is most commonly observed in elderly individuals with poorly controlled type 2 diabetes mellitus and is sometimes referred to as “diabetic striatopathy” or “non-ketotic hyperglycemic hemichorea-hemiballismus” (NH-HCHB) [1,2]. The condition is frequently misdiagnosed as a cerebrovascular event due to its acute presentation. Neuroimaging can help distinguish HCHB, often showing hyperdensity in the contralateral basal ganglia on CT scans [3-5].

The exact mechanism underlying NH-HCHB remains unclear, but several theories have been proposed, including metabolic disturbances, perfusion abnormalities, and alterations in neurotransmitter activity within the basal ganglia [4,6,7]. While most patients experience complete resolution of symptoms with proper glycemic control, some cases demonstrate persistent or recurrent involuntary movements, particularly when blood glucose remains poorly controlled [7-9]. Rare presentations, including atypical progression or bilateral involvement, have also been reported [9,10].

## Case presentation

A male in his late 80s presented to the emergency department with a fall. He was noted to have involuntary movement with no associated weakness and a focal neurologic deficit. He was worked up for stroke, had an MRI for transient ischemic attack, which did not show any acute findings, and self-discharged.

He presented three days later with progressive involuntary movements, initially affecting the left upper limb and later involving the left leg, lips, and tongue. He has a background of type 2 diabetes mellitus, hypertension, and chronic kidney disease. He has no known family history of movement disorder. He was not compliant with oral hypoglycemic agents or with insulin in the community.

On examination, there was a hyperkinetic movement noted on the upper and lower sides of the left limb (chorea). He also has a repetitive kicking movement on the left leg (ballismus) with tongue twitching and tongue deviation (athetosis). No other focal neurological deficits were noted.

Prior to the onset of his HCHB symptoms, he was walking with a stick to mobilize, with his wife assisting with care. He is an ex-smoker and denies any alcohol use.

Laboratory

His inflammatory markers were normal. He had glucose of 27 mmol/l, ketones of 0.0 mmol/l, and HbA1c of 175 mmol/mol. We have noted very poor glucose control with increasing HbA1c in the last four months. Initial serum sodium was 145 mmol/L (reference: 135-145 mmol/L), bicarbonate 24 mmol/L (22-29 mmol/L), pH 7.38, and anion gap 10 mmol/L (8-16 mmol/L). Calculated serum osmolality was 315 mOsm/kg (normal: 275-295 mOsm/kg) (Table 1).

**Table 1:** Summary of HbA1c, glucose, ketones, and CRP blood results HbA1c: glycated hemoglobin, CRP: C-reactive protein

Components	28/02/2025	21/01/2025	25/10/2024	01/10/2024	Reference values
HbA1c (mmol/mol)	175	121	70	59	20-41 (mmol/mol)
Glucose (mmol/ml)	27	14.3	11.2	5.0	4.0-5.4 (mmol/ml)
Ketones (mmol/ml)	0.0	0.0	0.0	0.0	<0.6 (mmol/ml)
CRP (mg/ml)	0.6	0.8	<0.6	<0.6	<1 (mg/ml)

Imaging

**Figure 1:**
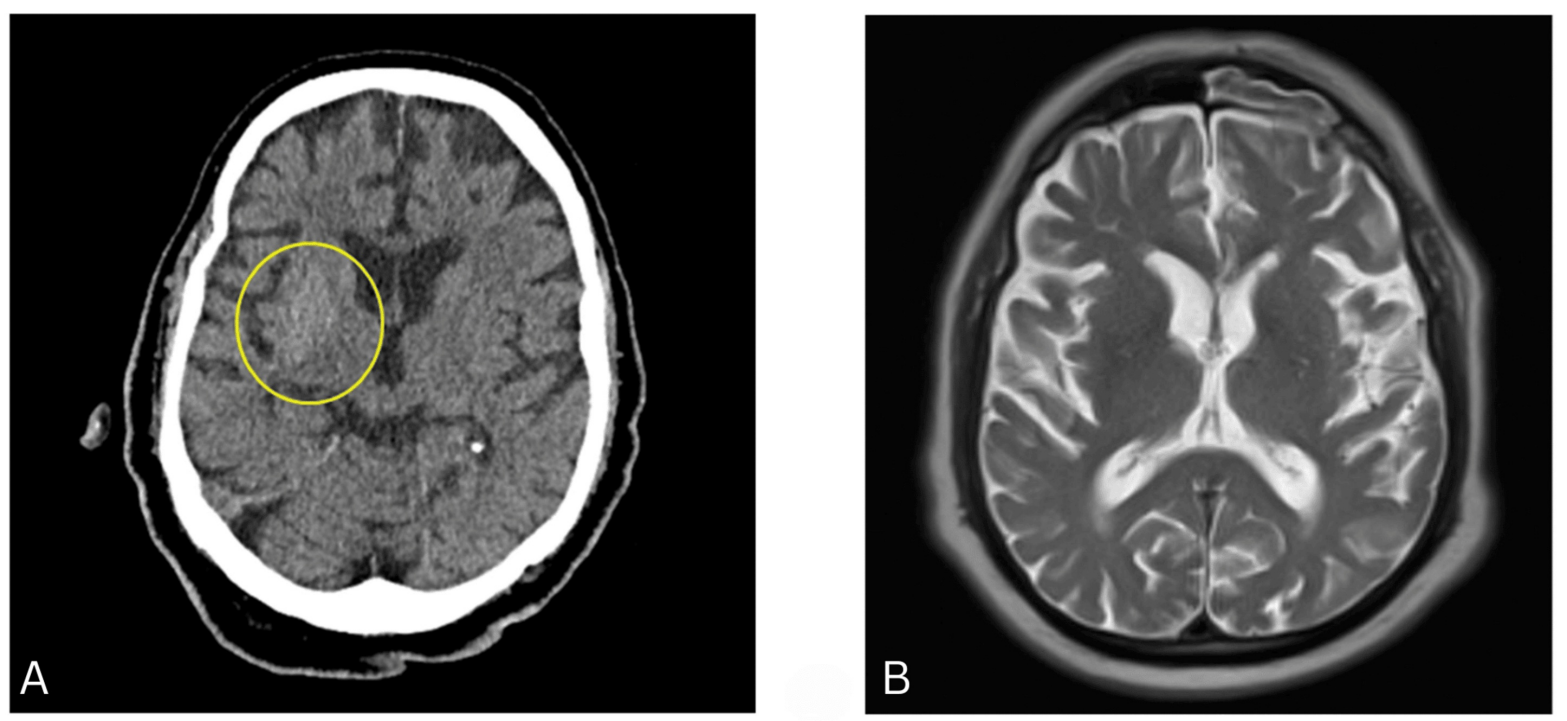
Non-contrast CT head scan (A) and T2 weighted MRI head scan (B) conducted one week apart (axial view) CT: computed tomography, MRI: magnetic resonance imaging

A non-contrast CT head scan demonstrated hyperdensity in the right lentiform and caudate nuclei (Figure 1A). Axial T2-weighted MRI ruled out hemorrhagic stroke, infarct, post-stroke movement disorder, and structural basal ganglia lesions (Figure 1B). Interestingly, the hyperdensity seen on the right lentiform was not evident on the MRI performed one week later [10,11].

He was seen by the diabetic team and was started on insulin Novomix 30 BD. The neurology team started symptomatic treatment with low-dose risperidone (0.5 mg once daily), which was ineffective, followed by a cautious switch to haloperidol, which was titrated gradually to 3 mg twice daily for symptomatic control. Haloperidol was selected due to its established efficacy in hyperkinetic movement disorders, including HCHB, particularly when glycemic optimization alone does not achieve symptomatic resolution.

Given the patient’s advanced age and chronic kidney disease, potential risks of QT prolongation, extrapyramidal effects, and sedation were carefully weighed and discussed with the patient and family. Baseline and follow-up ECGs were performed, and QTc intervals remained within normal limits throughout treatment.

Trials of VMAT2 inhibitors (tetrabenazine) and clonazepam were attempted but poorly tolerated, leading to excessive somnolence, confusion, and poor compliance. These agents were therefore discontinued. Haloperidol was continued as the most effective and tolerable option in this patient’s clinical context. Despite significant initial improvement in hemiballismus, mild residual choreiform movements persisted.

Outcome and follow-up

The hemiballismus showed significant initial improvement with haloperidol, but chorea persisted. At the two-week outpatient review, he had persistent choreiform movements despite improved glycemic control (BM 8-12 mmol/L).

After three months, the patient developed new right-arm spasms, poor sleep, and intermittent visual hallucinations. These symptoms raised concern for disease progression, neuroleptic side effects, or an intercurrent metabolic or infectious pathology. A repeated metabolic workup excluded acute infection or electrolyte imbalance, suggesting a multifactorial cause related to ongoing basal ganglia dysfunction and potential medication effects.

His long-term sugar levels were difficult to control in the community due to compliance issues, reduced renal function, and needle phobia. He was noted to have a worsening of abnormal movement symptoms with raised sugar levels.

A follow-up MRI was planned to assess for structural or metabolic evolution of the basal ganglia findings and to correlate with the patient’s persistent choreiform symptoms. However, the scan was not completed, as the patient demonstrated poor compliance with outpatient follow-up and subsequently missed scheduled imaging appointments. Over the following months, his clinical condition deteriorated, and he was transitioned to palliative care. At that point, further neuroimaging was not pursued in accordance with comfort-focused goals of care.

Due to the inability to optimize sugar levels in the community and further deterioration in health, the patient is now requiring 24-hour care, which is a significant change in his quality of life. He was meant to follow up at the OP neurology clinic; however, six months post-admission, he deteriorated further and is now under the palliative team for comfort.

## Discussion

NH-HCHB is a rare but important differential diagnosis for acute involuntary movements in elderly diabetic patients [1-3]. It typically presents with contralateral hyperkinetic movements and characteristic radiological changes in the basal ganglia. The pathophysiology is thought to involve metabolic disruption in GABAergic pathways due to hyperglycemia-induced perfusion abnormalities [3,4]. While presentation is mostly unilateral, bilateral involvement has been reported [5,7]. Imaging may not always confirm the diagnosis, highlighting the importance of clinical correlation with history, examination, and laboratory findings [12].

Management generally includes strict glycemic control and neuroleptic therapy, with symptom resolution in most cases [1-4]. However, patient-specific factors, comorbidities, and social circumstances can complicate treatment, as seen in this case where persistent and progressive symptoms occurred despite optimized diabetic and neuroleptic therapy [5-7]. Early family discussion and multidisciplinary planning are essential to guide care and support long-term outcomes [6,9,11].

## Conclusions

This case illustrates the complexity of managing NH-HCHB, especially in elderly patients with multiple comorbidities and poor glycemic control. Although NH-HCHB is generally reversible with timely glucose regulation and neuroleptic therapy, this patient’s course was atypical, with persistent and progressive choreiform movements. The case underscores the importance of early recognition of NH-HCHB in diabetic patients presenting with unilateral involuntary movements. It highlights the need for multidisciplinary involvement to address both medical and social challenges, including treatment compliance and long-term care planning. Ultimately, it demonstrates that while NH-HCHB is often treatable, patient-specific factors can significantly influence outcomes, emphasizing the necessity of a personalized, holistic approach.
